# Population Pharmacodynamics of IPX066: An Oral Extended-Release Capsule Formulation of Carbidopa–Levodopa, and Immediate-Release Carbidopa–Levodopa in Patients With Advanced Parkinson’s Disease

**DOI:** 10.1002/jcph.63

**Published:** 2013-02-20

**Authors:** Zhongping Mao, Ann Hsu, Suneel Gupta, Nishit B Modi

**Affiliations:** Impax PharmaceuticalsHayward, CA, USA

**Keywords:** IPX066, levodopa, Parkinson’s disease, pharmacodynamics

## Abstract

A pharmacodynamic model is presented to describe the motor effects (tapping rate, Unified Parkinson’s Disease Rating Scale [UPDRS] Part III, and investigator-rating of ON/OFF, including dyskinesia) of levodopa (LD) in patients with advanced idiopathic Parkinson’s disease (PD) treated with immediate-release (IR) carbidopa–levodopa (CD–LD) or an extended-release (ER) formulation of CD–LD (IPX066). Twenty-seven patients participated in this open-label, randomized, single-and multiple-dose, crossover study. The pharmacodynamic models included a biophase effect site with a sigmoid E_max_ transduction for tapping and UPDRS and an ordered categorical model for dyskinesia. The pharmacodynamics of LD was characterized by a conduction function with a half-life of 0.59 hours for tapping rate, and 0.4 hours for UPDRS Part III and dyskinesia. The LD concentration for half-maximal effect was 1530 ng/mL, 810 ng/mL, and 600 ng/mL for tapping rate, UPDRS Part III, and dyskinesia, respectively. The sigmoidicity of the transduction was 1.53, 2.5, and 2.1 for tapping rate, UPDRS Part III, and dyskinesia, respectively. External validation of the pharmacodynamic model using tapping rate indicated good performance of the model.

Parkinson’s disease (PD) is a progressive neurodegenerative disease that affects approximately 1–2% of the population above 60 years of age.^1^ The cardinal clinical manifestations of PD are resting tremor, rigidity, bradykinesia, and gait dysfunction. During the early stages of the disease, about 70% of patients may experience a slight tremor. Bradykinesia is described as a general reduction in spontaneous movement, and can cause difficulty with repetitive movements, such as finger tapping. Rigidity may cause stiffness of the limbs, neck, and trunk and may affect walking. The Unified Parkinson’s disease rating scale (UPDRS) is a scale developed by the Movement Disorders Society Task Force on Rating Scales for Parkinson’s Disease.^2^ This scale provides an efficient and flexible means of monitoring PD-related disability and impairment, and has been used in studies of early, mild, moderate, and advanced disease with motor fluctuations. The total UPDRS scale comprises four components: Part I, Mentation, Behavior, and Mood; Part II, Activities of Daily Living; Part III, Motor; Part IV, Complications of therapy.

Levodopa (LD), combined with a dopa decarboxylase inhibitor such as carbidopa (CD) or benserazide, continues to be an important mainstay in the symptomatic treatment of PD. No other medicinal or surgical therapy currently available has been shown to provide antiparkinsonian benefits superior to those achieved with LD.^3^–^5^ During the early stages of the disease, immediate release (IR) LD provides a stable response and has an adequate duration of effect. However, as the disease progresses and with continued LD therapy, the duration of effect from each dose progressively decreases and begins to approximate the plasma half-life of the drug. Eventually, patients may fluctuate between periods of “ON” when the subject’s medication is providing benefit with regard to mobility, slowness, stiffness, and periods of “OFF” which is defined as the state when the medication has worn off and is no longer providing benefit. A majority of patients treated with LD will experience motor fluctuations, dyskinesia or other complications within 5 years of treatment. Dyskinesias are involuntary twisting, turning movements which are an effect of medication and occur during the “ON” state. Non-troublesome dyskinesias do not interfere with function or cause meaningful discomfort. Troublesome dyskinesias interfere with function or cause meaningful discomfort.

Although several controlled-release (CR) formulations of LD (with CD or benserazide) have been developed, these formulations are associated with erratic absorption and variable LD plasma concentrations.^6^–^8^ In addition, the latency to onset of motor improvement is typically 30–90 minutes for the IR formulation and 60–180 minutes with the CR formulation due to the slower absorption.^3^,^9^,^10^ Thus, CR CD–LD is commonly administered with IR CD–LD in patients with fluctuations to improve the control of PD symptoms, particularly for the first dose in the morning.^9^–^12^

IPX066 is a multiparticulate extended-release (ER) oral capsule formulation of CD–LD. The CD:LD ratio is 1:4 similar to current CD–LD products. The formulation is designed to provide a rapid initial absorption of LD to achieve therapeutic concentrations of LD and maintain these therapeutic concentrations for an extended duration to assure an early “on” state and sustained efficacy. Pharmacokinetic properties of this formulation and the associated efficacy in patients have been reported previously.^13^ This report focuses on the development of population pharmacodynamic models for IPX066 and IR CD–LD in patients with advanced idiopathic PD.

## Methods

### Study Design

The design of this Phase 2 study has been reported previously.^13^ Briefly, this was an open-label, multicenter, randomized, two-period, two-treatment, single-and multiple-dose, crossover study comparing IPX066 and IR CD–LD in patients with idiopathic PD with motor fluctuations. The protocol was approved by the investigational review boards of the participating institutions, subjects provided informed consent, and the study was conducted in accordance with ethical principles that are consistent with good clinical practice. Subjects were randomized to one of two dosing sequences: IPX066 followed by IR CD–LD or IR CD–LD followed by IPX066. During each treatment period, subjects received 8 days of medication. During the IR CD–LD treatment, patients continued therapy according to their prestudy LD regimen, with dose adjustments for excluded COMT inhibitors and other LD formulations. Subjects were assigned their initial IPX066 regimen by the investigator based on a dosage conversion table, with IPX066 dosed approximately every 6 hours. The suggested initial conversion for IPX066 was designed to achieve similar peak concentrations of LD for the IPX066 and IR CD–LD regimens. Pharmacokinetic and efficacy/pharmacodynamic measurements were assessed in the clinic following a single dose (Day 1) and following multiple dosing (Day 8) of each treatment period. Tapping speed, a surrogate measure of bradykinesia,^14^ was measured by the number of times in 1 minute the subjects could alternatively tap two counter keys 20 cm apart with the most affected arm. Walking speed was assessed as the time required to rise from a chair, walk 6 m, turn, and to return to a sitting position. On Day 1, tapping and walking assessments were conducted every 30 minutes, beginning 1 hour before and for 8 hours after dosing. On Day 8, assessments were conducted for 12 hours. Motor function was assessed using the Motor Examination portion (Part III; 14 questions) of the UPDRS. Each subject also completed a PD diary using the Hauser diary^15^ on three consecutive days immediately prior to the first treatment and at the end of each treatment period (Days 5, 6, and 7). In addition, the investigator staff evaluated each subject every 30 minutes on Day 1 and every hour on Day 8 during the clinic visits to assess the degree of motor effect (ON/OFF) and dyskinesia. In order to maintain similar predose conditions between the two treatment periods on Day 1, subjects returned to their prestudy anti-PD regimens for approximately 1 week between the two treatment periods, and stopped taking CD–LD after 10:00 pm prior to the Day 1 clinic dose of each treatment period. Prior to the Day 8 visits of each treatment period, subjects were allowed to take study medications as needed. Since several patients were unable to initiate or complete the timed walk, the pharmacodynamic modeling focused on tapping rate, UPDRS Part III, and investigator-rating of ON/OFF, including dyskinesia.

### Tapping and UPDRS Modeling

Studies have suggested a time delay between the plasma LD concentration and pharmacodynamic response.^16^–^18^ This time delay may be modeled by linking the plasma concentration to a biophase effect site concentration, c_e_(t), by means of a conduction function,

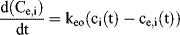
(1)
where c_i_(t) is the observed plasma LD concentration at time t for the i-th individual, and k_eo_ is the first order rate constant that governs the exit of drug from the biophase.

Several authors have suggested that the clinical response in PD is related to the LD effect site concentrations by a sigmoid relationship.^19^–^22^ Tapping rate was modeled as the baseline tapping rate (E_0_) plus the drug effect following treatment using a sigmoid E_max_ transduction function

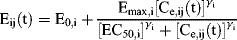
(2)
where E_ij_ is the j-th effect measurement for the i-th individual and E_0,i_ is the observed baseline tapping rate for the i-th subject. The sigmoid E_max_ transduction function has parameters E_max_, EC_50_ and γ, where E_max_ is the maximum increase in tapping rate due to drug, EC_50_ is the biophase concentration that results in 50% of the maximum effect, and γ is the Hill coefficient, which describes the shape and steepness of the concentration–effect relationship.

In the case of the UPDRS Part III score, the maximum increase in response was modeled as proportional to the baseline score,

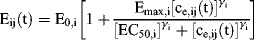
(3)

The inter-individual variability for all parameters was modeled using an exponential error model and residual error was modeled as proportional.

### Dyskinesia Modeling

Investigator-rating of ON/OFF and dyskinesia was modeled as an ordered categorical response using a method described by Mandema and Stanski.^23^ The categorical response was described as 1 = “off/asleep,” 2 = “on without dyskinesia,” 3 = “on with non-troublesome dyskinesia,” and 4 = “on with troublesome dyskinesia.” If a subject was sleeping they were considered “off” and assigned a score of 1. If PD_ij_ is the dyskinesia rating score of patient i at time j, the probability (Pr) that PD_ij_ ≥ k, k 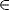 (1, 2, 3, 4), can be modeled as:

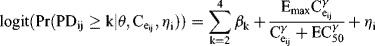
(4)
where logit denotes the logistic function of the probability (Pr); θ = {β, k_eo_, E_max_, EC_50_} for various degrees of dyskinesia (there is no estimate for β_1_ since Pr(PD ≥ 1) = 1); C_e_ denotes the effect-site concentration given by Equation (1) and η represents the inter-subject variability. The probability of dyskinesia PD_ij_ = k, k{1, 2, 3, 4}, is given as:

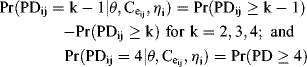
(5)

The joint likelihood (L) is the product of the probability observed at each data point with the product of the likelihood of the random effects,

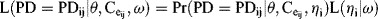
(6)
where ω is the square root of the variance of the random effects to be estimated. Assuming that the dyskinesia rating of patient i at distinct times j are independent, the likelihood across all data points is given as:

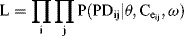
(7)

### Covariate Model Selection

The potential impact of formulation (IPX066 vs. IR CD–LD) and treatment day (Day 1 vs. Day 8) were examined. In addition, Hoehn and Yahr stage was examined as a categorical covariate for tapping rate and for UPDRS Part III. Additional covariates, such as gender and race, were not examined since this was a small study (n = 27 subjects) and a majority of the subjects were males (78%) and Caucasian (89%). Significance of the covariates was ascertained at the 0.01 level using a log-likelihood criterion.

### Model Validation

The exposure response model for the continuous pharmacodynamics endpoints tapping rate and UPDRS Part III was validated using a non-parametric bootstrap analysis. Parameters from 500 replicate trials were calculated by re-sampling the dataset. Medians and 95% confidence intervals (CI) of the bootstrap parameters were compared with the estimated values and 95% CI of the final model.

The pharmacodynamic model for tapping rate was validated externally using data from a separate four-period, randomized crossover study that evaluated two doses of a different modified-release bilayer tablet formulation of CD–LD (IPX054, Impax Laboratories, Inc.), IR CD–LD (Sinemet®), and CR CD–LD (Sinemet CR®) in a group of 16 subjects with advanced PD. In this study, subjects were treated with single doses of IPX054 200 mg, IPX054 250 mg, IR CD–LD 200 mg or CR CD–LD 200 mg in a randomized, crossover fashion. LD plasma concentrations were measured frequently and tapping rates were measured every 30 minutes beginning 1 hour predose and continuing for 8 hours postdose.

### Software

Parameter estimation was done using nonlinear mixed effects modeling (NONMEM, Version 7.1, ICON Development Solutions, Ellicott City, MD). Whenever practical, the first-order conditional estimation method with interaction (FOCE INTER) was used. Otherwise, the first-order (FO) estimation method was used. Non-parametric bootstrap analysis was done using Wings for NONMEM. Visual evaluation of the models and simulations were conducting using R 2.9.1.

## Results

Twenty-seven subjects were enrolled and completed the study. Demographic and baseline characteristics of the patients have been reported previously.^13^ Briefly, the subjects were predominantly male (78%) and white (89%), with 85% at Hoehn and Yahr Stage 2 or 3.

### Tapping

The estimated model parameters are summarized in Table.1. The estimated maximum increase in tapping rate was 93.7 taps/minute, EC_50_ was 1590 ng/mL and the effect compartment equilibrium rate constant, k_eo_, was 1.17 hour^−1^, corresponding to an equilibrium half-life of 0.59 hour, and the Hill coefficient was 1.53. The non-parametric bootstrap analysis results matched with the estimates of the model parameters. The median and 95% CIs of the bootstrap estimates compared favorably with the estimated parameters for the original data set. The effect of treatment (IPX066 or IR CD–LD) and treatment day (Day 1 or Day 8) on the model parameters was examined, and no statistically significant effect was noted for E_max_ or EC_50_, hence a single model described the pharmacodynamics for both IPX066 and IR CD–LD.

**Table:** Pharmacodynamic Model Parameter Estimates for Tapping and UPDRS Part III

Parameter	Maximum Likelihood Estimate (95% CI)	Bootstrap Median (95% CI)
Tapping		
E_max_ (Taps/min)	93.7 (84.4, 104)	79.8 (47.4, 126.6)
EC_50_ (ng/mL)	1590 (1210, 2080)	1380 (620, 3605)
k_eo_ (1/h)	1.17 (0.87, 1.54)	1.37 (0.98, 1.78)
γ	1.53 (1.27, 1.86)	1.60 (0.94, 3.26)
ωlog(E_max_) (%)	83.2	
ωlog(k_eo_) (%)	65.3	
ωlog(γ) (%)	113	
UPDRS Part III		
E_0_	31.8 (29.8, 34.0)	32.7 (25.0, 38.5)
E_max_ (% change from baseline)	63 (56, 70)	66 (54, 145)
EC_50_ (ng/mL)	812 (672, 982)	804 (620, 3967)
k_eo_ (1/h)	1.80 (1.24, 2.59)	1.83 (1.15, 2.92)
γ	2.5 (1.7, 3.5)	2.1 (0.68, 3.5)
ωE_0_ (%)	19.3	
ωlog (E_max_; %)	55.5	
ωlog (EC_50_; %)	101	
ωlog (k_eo_; %)	90.3	
ωlog(γ; %)	85.6	

**Figure 1:**
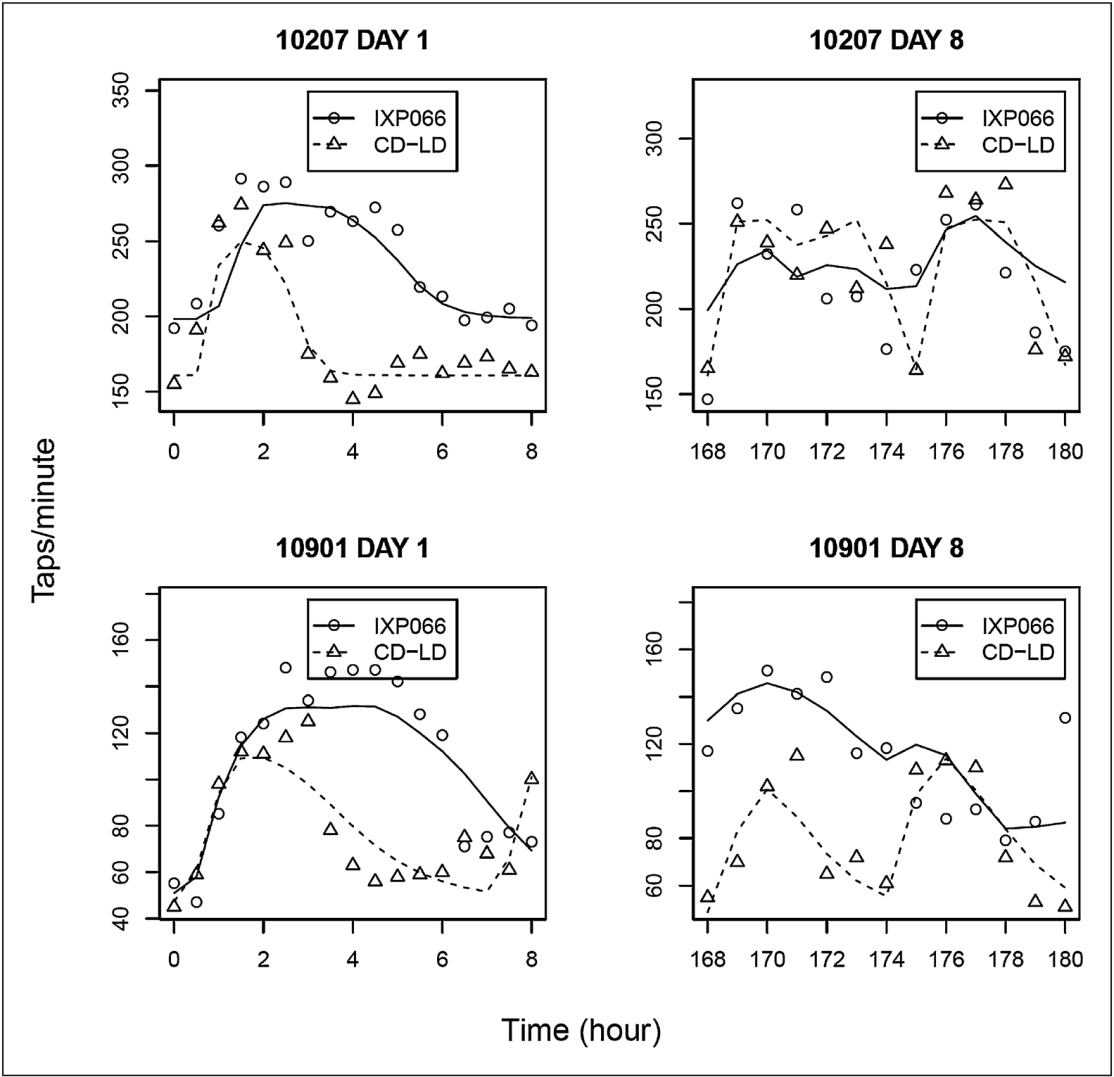
Observed time course of tapping (IPX066, diamond; IR CD–LD, triangle) in two representative subjects following single (Day 1) and multiple (Day 8) dosing. The lines represent the fitted profiles (IPX066, solid line; IR CD–LD, dashed line).

The sigmoid E_max_ model describes the tapping response well for both IPX066 and IR CD–LD following single (Day 1) and multiple dosing (Day 8). The adequacy of the model was judged by its ability to describe the time course of tapping rate for individual subjects. This is illustrated in Figure 1 for two representative subjects. Visual predictive check indicated good agreement between the predicted and observed mean profile and the 95% prediction interval generally encompassed 95% of the observations for both treatments following single or multiple dosing (Supplementary Materials Figure 1). On Day 1, when subjects received a single dose of IPX066 or IR CD–LD, the onset of effect after IPX066 was comparable to that for IR CD–LD and the duration of effect was longer for IPX066 than for IR CD–LD. The magnitude of effect following IPX066 was greater than that following IR CD–LD and the baseline effect on Day 8 was higher with IPX066 than with IR CD–LD.

### UPDRS Part III

The percent change in UPDRS Part III score was modeled as a sigmoid E_max_ response to the effect compartment LD concentration. The estimated parameters of the final model are presented in Table 1. The estimated baseline UPDRS Part III score was 31.8. The estimated maximum reduction in UPDRS Part III score from baseline was 63% of the baseline value, effect site EC_50_ was 812 ng/mL, and k_eo_ was 1.80 hour^−1^, corresponding to an equilibrium half-life of 0.39 hour. The parameters estimated using a bootstrap approach compared favorably with the model estimates.

**Figure 2:**
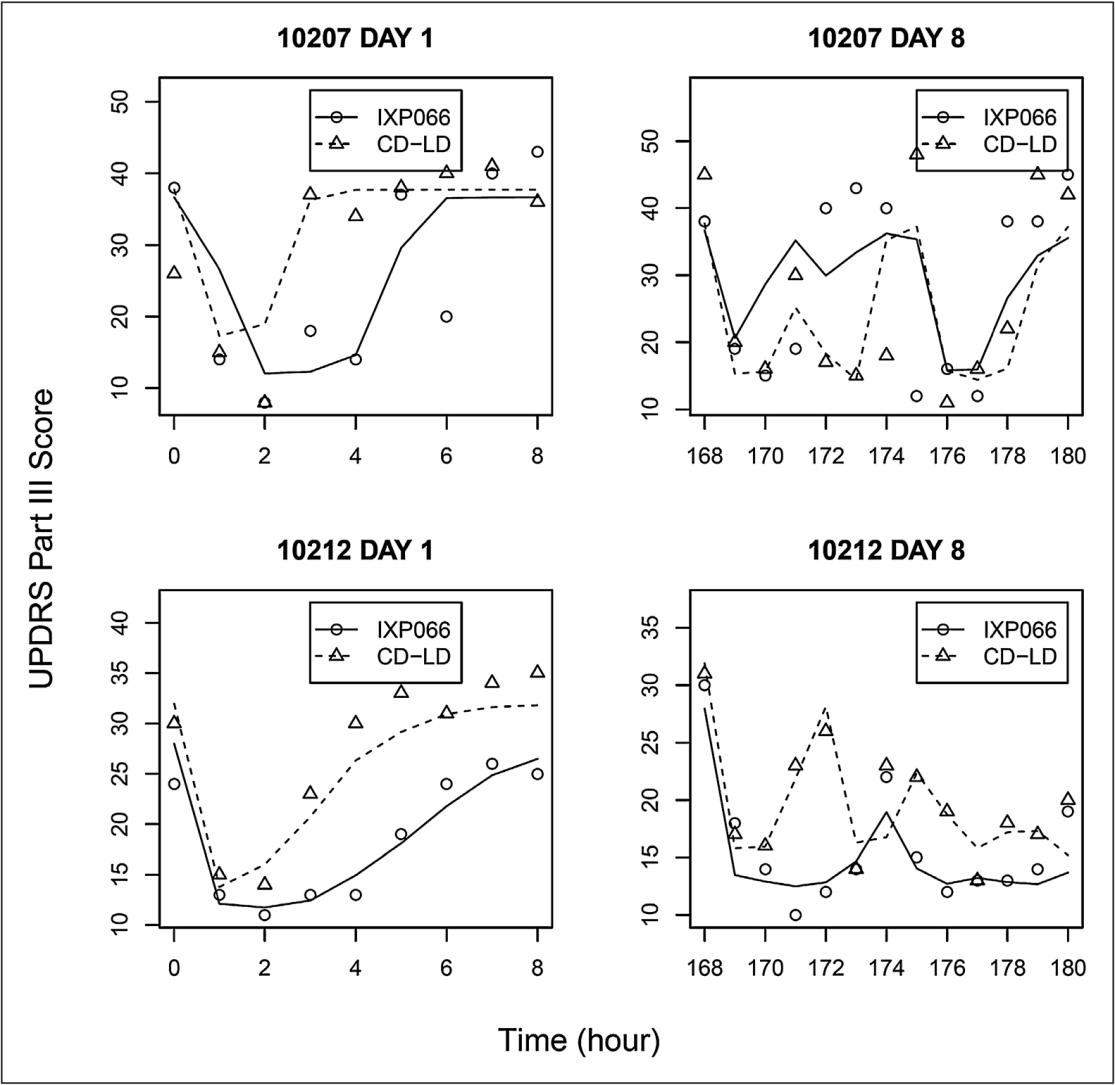
Observed time course of UPDRS Part III (IPX066, diamond; IR CD–LD, triangle) in two representative subjects following single (Day 1) and multiple (Day 8) dosing. The lines represent the fitted profiles (IPX066, solid line; IR CD–LD, dashed line).

The inhibitory sigmoid E_max_ model adequately described the UPDRS Part III data following single and multiple dosing of IPX066 and IR CD–LD. The predicted mean response described the observed data and the 95% prediction band encompassed the majority of the observed data (Supplementary Materials Figure 2). The model adequately described the observed UPDRS Part III time course for individual subjects as illustrated in Figure 2 for two representative subjects. On Day 1, the onset of effect after a single dose of IPX066 is comparable to that of IR CD–LD, while the effect for IPX066 lasted longer. On Day 8, the change in UPDRS Part III score for subjects during IPX066 treatment is greater and more constant than during IR CD–LD treatment.

### Dyskinesia

The estimated parameters of the final model for investigator rating of ON/OFF and dyskinesia are presented in Table 2. The estimated EC_50_ was 604 ng/mL and k_eo_ was 1.55 hour^−1^, corresponding to an equilibrium half-life of 0.45 hour.

**Table:** Dyskinesia Model Parameters

Parameter	Model Estimate (95% CI)
β1	1 (Fixed)
β2	−3.91 (−5.16, −2.66)
β3	−2.48 (−3.61, −1.35)
β4	−3.14 (−4.29, −1.99)
k_eo_ (h^−1^)	1.55 (1.06, 2.28)
E_max_	7.32 (5.79, 9.23)
EC_50_ (ng/mL)	601 (471, 769)
γ	2.1 (1.5, 3.0)
ω _(β3)_	0.96
ω (log(E_max_))	0.52

**Figure 3:**
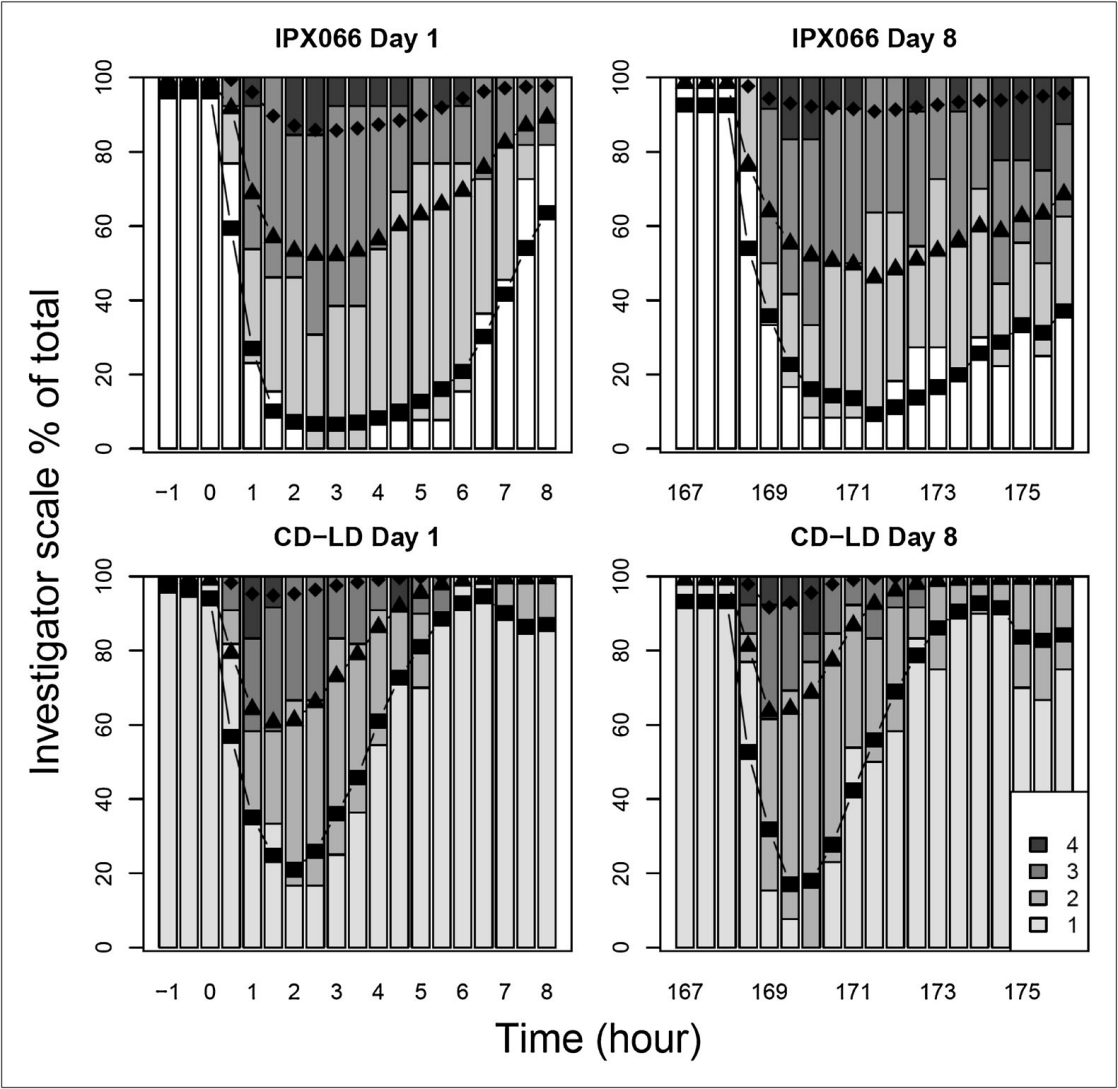
Comparison of Observed and Predicted Investigator Rating Scale in patients treated by IPX066 (upper panel) and IR CD–LD (lower panel). Bars represent observed data with scores of 1 (off/asleep; lightest), 2 (on without dyskinesia), 3 (on with non-troublesome dyskinesia), and 4 (on with troublesome dyskinesia; darkest). Solid lines with symbols represent the predicted responses.

The time course of the observed and model predicted probability of the investigator-rating scores for IPX066 and IR CD–LD on Day 1 and Day 8 is illustrated in Figure 3. There was a greater reduction in the probability of subjects being “OFF” and a corresponding higher probability of being “ON” with IPX066 than with IR CD–LD. In addition, consistent with the sustained LD concentration profile from IPX066, the duration of patients being “ON” was also longer for IPX066. The effect site LD concentration–investigator rating scale relationship for the categorical model is illustrated in Supplementary Materials Figure 3.

### Model Validation

Data from a separate four-period crossover study were used to externally validate the pharmacodynamic model for tapping rate. Tapping rate following the four treatments included in this study were simulated using the observed LD plasma concentration and the estimated pharmacodynamic parameters for the tapping model from the current study. The mean predicted tapping rates and the 5th–95th percentile prediction bands appeared to closely match the observed data, suggesting that the tapping model was robust. The predicted tapping rate along with the observed pharmacodynamic responses for the four treatments is presented in Supplementary Materials Figure 4.

## Discussion

A population pharmacodynamic model was developed to describe the single-and multiple-dose response to two formulations of CD–LD that result in different plasma profiles of LD. The pharmacodynamic responses evaluated in the study are endpoints that are commonly used to evaluate the effect of LD in PD. Tapping speed is an objective, quantitative measure of dexterity that has been used as a surrogate measure of bradykinesia, one of the cardinal motor symptoms of PD. The UPDRS Part III provides an efficient means of quantitatively assessing PD-related motor disability and impairment by the clinician. This endpoint has been used in studies of early, mild, moderate, and advanced disease with motor fluctuations. In addition, the study included investigator assessments of “OFF,” “ON,” and dyskinesia, which are direct outcomes of LD therapy.

The pharmacodynamic parameters estimated in the present study for tapping rate (E_max_ 93.7 taps/minute; EC_50_ 1590 ng/mL, k_eo_ 1.80 hour^−1^) are comparable to those reported by Nelson et al.^24^ who reported an E_max_ of 93.3 taps/minute and an EC_50_ of 1.6 mcg/mL in a study characterizing the pharmacodynamics of CR CD–LD (Sinemet® CR4) in PD patients. Similarly, Triggs et al.^21^ reported equilibrium half-life values of 173 and 43.3 minutes, and EC_50_ values of 0.35 and 1.4 mcg/mL in patients with Hoehn and Yahr scores of 1 and 4, respectively.

The pharmacodynamic parameters estimated in the present study for UPDRS Part III (E_max_ 63% of E_0_; EC_50_ 812 ng/mL; k_eo_ 1.80 hour^−1^; γ 2.5) are comparable to those reported by Trocóniz et al.^22^ (E_max_ 49% of E_0_; EC_50_ 951 ng/mL; k_eo_ 2.01 hour^−1^; γ 6.2). In a recent study, Adamiak et al.^25^ evaluated the pharmacodynamic effects (UPDRS Part III) of LD in 14 patients with advanced PD at Hoehn and Yahr Stage 2 and 3. In their study, these investigators estimated an E_max_ value of 23 and 29 for patients at Hoehn and Yahr Stage 2 and 3, respectively. Estimated values of EC_50_ were 601 ng/mL and 756 ng/mL, respectively. These values are comparable to those noted in the present study. However, Adamiak and colleagues reported Hill coefficient values of 4 and 9 for patients at Hoehn and Yahr Stage 2 and 3, respectively. The Hill coefficients reported by these authors were larger than that noted in the present study or those reported by Trocóniz.^22^ Both the present analysis and that by Trocóniz^22^ employed a mixed effect population approach whereas Adamiak et al.^25^ used a two stage approach, which may have contributed to the wider variability in estimation of the Hill coefficient. However, all these models suggest a steep exposure–response relationship.

Comparison of the pharmacodynamic parameters for tapping rate and UPDRS Part III indicates that both pharmacodynamic measures have generally similar equilibration rate constants (k_eo_) with half-lives of approximating 20 minutes and 40 minutes. These results are consistent with those reported with various other pharmacodynamic measures in PD (tapping, UPDRS, walking, sum of Columbia University Rating Scale).^26^

Table 3 presents the time to onset of effect and duration of effect for IPX066 and IR CD–LD estimated using the final pharmacodynamic parameter estimates (tapping and UPDRS Part III). A change of ≥15% from baseline in tapping rate and in UPDRS Part III score was defined as “ON” for efficacy. Previous studies have used a similar criterion of 10% or 15% change from baseline in defining effectiveness.^20^,^27^–^30^ The onset of treatment effect was rapid and generally comparable for IPX066 and IR CD–LD. The rapid onset of effect (22–23 minutes) noted with the tapping rate for IR CD–LD is consistent with that noted by others.^29^,^31^ In clinical practice, a standard dose of LD becomes effective within approximately 20 minutes.^32^ Both IPX066 and IR CD–LD have a similar initial increase in LD concentrations as measured by the time to reach 50% of C_max_ (0.76–0.78 hours in each case), which may explain the similar early onset of effect. The duration of effect was approximately 2 hours longer with IPX066 compared to IR CD–LD whether measured using tapping or UPDRS Part III. Consequently, the baseline effect on Day 8 was higher with IPX066 than with IR CD–LD, consistent with the ER properties of IPX066 (Supplementary Materials Figures 1 and 2). The longer duration of effect with IPX066 is consistent with the longer interval that LD concentrations are maintained above 50% of C_max_ with IPX066 compared with IR CD–LD (4.0 hours compared with 1.4 hours for IPX066 and IR CD–LD, respectively).^13^ As illustrated in Figures 1 and 2 for two representative subjects, the sustained LD concentrations with IPX066 also result in fewer fluctuations in the pharmacodynamic response compared to IR CD–LD.

**Table:** Comparison of Duration of Effect Using Tapping Rate and UPDRS Part III Estimated Using the Pharmacodynamic Models

Treatment	IPX066	IR CD–LD
Duration of effect (hours) on Day 1^a^		
Tapping	7.6	5.6
UPDRS Part III	7.4	4.5
Time to onset of effect (hours) on Day 1^a^		
Tapping	0.39	0.36
UPDRS Part III	0.61	0.46

a Change of more than 15% from baseline value.

Although the pharmacodynamic models for tapping and UPDRS Part III in the current analysis did not consider a circadian effect, this is unlikely to have a significant effect on the model. The current model assumed that the intrinsic tapping rate is constant across the day and that any potential diurnal effects could not be distinguished from the drug effect. There are conflicting reports on the diurnal response to oral LD. Frankel et al.^33^ evaluated the diurnal variation in the duration and magnitude of motor response to standard LD in PD. Motor response to LD did not differ significantly in the morning and afternoon, and the relationship between plasma LD concentrations and motor function was the same following morning and afternoon doses suggesting that there may not be a significant diurnal effect. LD PD models that have incorporated diurnal effects have involved modeling diurnal variation in the endogenous LD synthesis. The data have typically been from infusion studies involving multiple-day assessments with periods of LD administration and periods of no LD.^18^,^34^,^35^ The contribution of the diurnal variation in the endogenous LD synthesis to the pharmacodynamic effect has generally been small compared to the overall drug effect. Although Nutt et al.^34^ noted a significant decrease in tapping rate in the 15:00–18:00 and 19:00–22:00 time intervals compared to the 7:00–10:00 and 11:00–14:00 time intervals, the difference in mean tapping rate was approximately 30 taps/minute and these authors did not account for the potential effects of fatigue later in the day.

Although some previous studies have included tolerance in describing LD pharmacodynamics, the final UPDRS model in the present analysis characterizing the acute effects of LD did not include tolerance. This may be justified based on the fact that the subjects in the present study had been on a stable dose of CD–LD for an extended period of time prior to entry into the study and it is unlikely that tolerance would be noted over the 1-week study period. As a placebo treatment was not included in the current study, the dynamics of a placebo response cannot be identified. The current pharmacodynamic analysis also employed a single pharmacodynamic relationship for IPX066 and IR CD–LD in the final model. During model development, an examination was made to determine if inclusion of a treatment effect on the pharmacodynamic parameters improved the pharmacodynamic fits. Inclusion of treatment effects on E_max_ or EC_50_ resulted in no statistically significant decrease in the objective function, so a single pharmacodynamic relationship for IPX066 and IR CD–LD was employed in the final model.

The incidence of patients reporting troublesome dyskinesia in the present study was relatively low. Although the logit model adequately described the dyskinesia response, it assumes that the data are independent and identically distributed. It is likely that this may not be the case since the probability that a subject is in a given response state depends on what state they were in 30 minutes earlier. Mean daily doses of LD were 3054.4 and 816.7 for IPX066 and IR CD–LD, respectively, with peak LD plasma concentrations of 3000 ng/mL and 2360 ng/mL for IPX066 and IR CD–LD, respectively.^13^ Based on the concentration–effect relationship these concentrations are on the asymptote and hence may explain the lack of a difference in the degree of dyskinesia between IPX066 and IR CD–LD in spite of the higher LD dose with IPX066. Patients enrolled in the study were required to have been on a stable dose of CD–LD prior to their entry in the study, and hence likely to have been on a regimen designed to minimize troublesome dyskinesia.

The concentration–effect relationship for IPX066 is comparable to IR CD–LD for tapping and UPDRS Part III. IPX066 had an estimated onset of effect within approximately 20–40 minutes of administration, comparable to that noted with IR CD–LD. The estimated duration of effect for IPX066 was approximately 2–3 hours longer than that for IR CD–LD. The longer duration of effect is consistent with the sustained LD plasma-concentration profile from IPX066. The incidence of dyskinesia was low for both LD products.

The pharmacodynamic models indicate that the concentration–effect relationship for LD is similar for IPX066 and IR CD–LD. The rapid onset and longer duration of effect with IPX066 are a result of the LD concentration profile. The favorable pharmacodynamic effects noted with IPX066 in this short-term study in advanced PD patients merit further examination in longer term studies.

## Funding

This study was supported by Impax Pharmaceuticals, a division of Impax Laboratories, Inc. All authors are employees of Impax and may own stock or hold stock options in the company.
