# Supplementary material for: Population Pharmacodynamics of IPX066: An Oral Extended-Release Capsule Formulation of Carbidopa–Levodopa, and Immediate-Release Carbidopa–Levodopa in Patients With Advanced Parkinson’s Disease
Source: J Clin Pharmacol. 2013 Feb 20;53(5):523–31. doi: 10.1002/jcph.63 (PMC3798100; doi:10.1002/jcph.63)
Supplement: Supplementary file 1 [file jcph0053-0523-sd1.doc]

**Figure S1: Percentage Change from Baseline in Tapping following IPX066 and IR CD-LD on Days 1 and 8**

Symbols are mean observations with vertical bars representing the 95% confidence interval of the observation. Solid lines represent the mean prediction with the shaded area representing the 95% prediction interval.

**Figure S2: Percent Change from Baseline in UPDRS Part III Following IPX066 and IR CD-LD on Days 1 and 8**

Symbols are observations with the vertical bars representing 95% confidence interval. Solid lines represent the mean prediction with the shaded area encompassing the 95% prediction interval

**Figure S3: Concentration-Effect Relationship for the Categorical Model Describing Investigator Rating Scale (1=off/asleep; 2=on without dyskinesia; 3=on with non-troublesome dyskinesia; 4=on with troublesome dyskinesia).**

**Figure S4: External Validation of Tapping Model Showing a Comparison of the Observed and Predicted Responses**

Solid line represents the observed mean tapping response with the vertical bars representing 5 and 95th percentile. Dashed line is the mean predicted tapping response with the shaded band representing the 90% prediction interval.
